# Duck enteritis virus activates CaMKKβ-AMPK to trigger autophagy in duck embryo fibroblast cells via increased cytosolic calcium

**DOI:** 10.1186/s12985-018-1029-0

**Published:** 2018-08-06

**Authors:** Haichang Yin, Lili Zhao, Yiping Wang, Siqi Li, Hong Huo, Hongyan Chen

**Affiliations:** 1 State Key Laboratory of Veterinary Biotechnology, Heilongjiang Provincial Key Laboratory of Laboratory Animal and Comparative Medicine, Harbin Veterinary Research Institute, the Chinese Academy of Agriculture Sciences, 678 Haping Road, Harbin, 150069 People’s Republic of China; 2 College of Life Science and Agriculture Forestry, Qiqihar University, Qiqihar, 161006 China; 3 Heilongjiang Provincial Key Laboratory of Resistance Gene Engineering and Protection of Biodiversity in Cold Areas, Qiqihar, Heilongjiang 161006 China

**Keywords:** Duck enteritis virus, Autophagy, Cytosolic calcium, CaMKKβ, AMPK

## Abstract

**Background:**

The results of our previous study showed that impaired cellular energy metabolism contributes to duck enteritis virus-induced autophagy via the 5`-adenosine monophosphate-activated protein kinase (AMPK)/tuberous sclerosis complex 2/mammalian target of rapamycin pathway in duck embryo fibroblast (DEF) cells. However, it remains unknown whether any other underlying mechanisms of AMPK activation are involved in autophagy induction.

**Methods:**

The activity of CaMKKβ and AMPK in DEF cells infected with DEV were evaluated.The Effect of inhibitory activity of CaMKKβ on DEV-induced autophagy was investigated. In addtion to, the cytosolic calcium level in DEF cells infected with DEV were evaluated.The Effect of inhibitory cytosolic calcium level on DEV-induced autophagy was investigated.

**Results:**

In this study, duck enteritis virus (DEV) infection activated CaMKKβ and its substrate molecule AMPK at 36, 48, and 60 h post-infection (hpi). STO-609, a CaMKKβ inhibitor, or CaMKKβ siRNA significantly inhibited the activation of DEV to AMPK, LC3I to LC3II transformation, and GFP-LC3 puncta distribution. In addition, inhibition of CaMKKβ activity also significantly reduced progeny DEV titer and gB protein expression. Besides, cytosolic calcium (Ca^2+^) was higher in DEV-infected cells than mock controls at 36, 48, and 60 hpi, respectively. Treatment of DEV-infected cells with 1,2-Bis (2-aminophenoxy) ethane-N, N, N′, N-tetraacetic acid (BAPTA-AM) significantly reduced intracellular Ca^2+^ ion concentrations, as well as CaMKKβ and AMPK activities, and subsequent autophagy, in addition to viral protein synthesis and viral titer.

**Conclusions:**

These results showed that elevated [Ca^2+^]cyto-mediated activation of CaMKKβ managed the activation of AMPK, which then positively regulated autophagy, thereby providing further insight into DEV–host interactions.

## Background

Duck enteritis virus (DEV) is a member of the Herpesviridae family and has a typical morphology of herpesviruses and a double-stranded, linear DNA genome about 160 Kb in length. DEV can cause a variety of acute, septic, and highly fatal infectious diseases in water fowl, including duck viral enteritis (DVE), which is characterized by internal bleeding due to mucosal damage of the blood vessels and digestive tract, as well as lymphoid organ damage and lesion formation [1]. The occurrence of DVE was first reported in the Netherlands in 1923 and has since spread to other countries. In 1957, the occurrence of the DVE was first reported in China and has since prevailed in relatively developed areas in southern and eastern China [2]. DVE is widespread and spreads rapidly, resulting in high morbidity and mortality that results in huge economic losses to the duck industry. However, the relative lag in molecular biology research has restricted efforts in DVE prevention and control.

Virus infection can induce autophagy, which is a process of precise membrane-dependent regulation to ensure intracellular fluid balance [3–5]. This response can be either antiviral or promote virus replication, depending on the type of virus and the intracellular environment of the host cell [6, 7]. According to the type of substrate, species, regulatory mechanism, and transport process, autophagy includes macroautophagy, microautophagy, and molecular chaperone-mediated autophagy [8]. Macroautophagy commonly refers to the process of double layer membrane formation of autophagosomes by fusion of the endoplasmic reticulum (ER) with lysosomes. In this process, commonly referred to as autophagy, the contents of autophagosomes are degraded.

Calcium (Ca^2+^) is a ubiquitous intracellular messenger of some important signal transduction processes, including activation of enzymes, differentiation, proliferation, and gene transcription [9, 10]. Ca^2+^ and calcium-sensing proteins might play a dual role in the process of autophagy regulation, depending on the cell type, intracellular environment, and Ca^2+^ abundance [11, 12]. Excessive cytoplasmic calcium is released from the ER, and then was reported to induce autophagy through calcium/calmodulin-dependent protein kinase kinase-beta (CaMKK)-regulated activation of adenosine 5′-monophosphate-activated protein kinase (AMPK) and the subsequent inhibition of the activity of mammalian target of rapamycin (mTOR) [13, 14].

Some viruses can take advantage of Ca^2+^ ion-related pathways of the host to promote replication [15, 16], such as porcine circovirus type 2, which can induce the release of Ca^2+^ ions from the ER via the inositol 1,4,5-trisphosphate receptor in duck embryo fibroblast (DEF) cells, which is considered to be responsible for apoptosis induction [17]. Rotavirus coding of nonstructural protein 4, which releases Ca^2+^ ions into the cytoplasm, initiates autophagy to activate CaMKKβ signaling [18]. The hepatitis B virus X protein targets the human B-cell lymphoma 2 homolog to regulate CED-9, which induces an increase in cytoplasmic Ca^2+^ ion concentrations and subsequent cell death in *Caenorhabditis elegans* [19]. Herpes simplex virus triggers activation of calcium-signaling pathways [20], Elevated [Ca2+]cyto-mediated activation of CaMKKβexactly managed the activation of AMPK, which then positively regulated autophagy through suppressing mTOR in cells infected with Bluetongue virus [21].

Our previous studies showed that impaired cellular energy metabolism contributes to DEV-induced autophagy via the AMPK/TSC2/mTOR pathway in DEF cells [22, 23]. However, it remains unknown whether other underlying mechanisms of AMPK participate in autophagy induction. The results of the present study demonstrated that CaMKKβ is an upstream regulator of AMPK during DEV infection, which contributes to autophagy induction. Activation of CaMKKβ results from an increase in cytosolic Ca^2+^ content. This research lays a foundation for DEV pathogenic mechanism research and provides further insight into DEV–host cell interactions.

## Methods

### Cells, viruses, and plasmids

DEF cells were obtained from 9 to 11 days specific pathogen-free duck embryos, as described previously [24], and cultured in Dulbecco’s modified Eagle’s medium (cat. no. 8116176; Gibco, Grand Island, NY, USA) supplemented with 5% fetal bovine serum (cat. no. 1722658; Gibco) and antibiotics (0.1 mg/ml of streptomycin and 0.1 mg/ml penicillin) at 37 °C under an atmosphere of 5% CO_2_/95% air. DEV strain CSC was kept in our laboratory.

To construct a GFP-LC3 recombination plasmid, the LC3 gene was amplified from DEF cells with the primer pair LC3F 5`-ATG CAA CCG CCT CTG-3` and LC3R 5`-TCG CGT TGG AAG GCA AAT C-3`, corresponding to the GenBank sequence for duck LC3B gene (NW_004676873.1), and cloned into the pEGFP-C1 vector, to express LC3B protein with the GFP protein.

### Virus infection and drug or small interfering RNA (siRNA) treatment

DEF cells were infected with DEV for 2 h at 37 °C, washed three times with sterile phosphate-buffered saline (pH 7.4), then maintained in 2% in culture medium supplemented with fetal bovine serum for various time points until samples were harvested. The cells were then cultured in 2% culture medium supplemented with fetal bovine serum with or without pre-treatment with the same drug for the indicated times. The optimal concentrations of chemicals used in this experiment were 10 mM 1,2-Bis(2-aminophenoxy)ethane-N,N,N′,N-tetraacetic acid (BAPTA-AM; Abcam, Cambridge, UK), 5 μM STO-609 (Merck-Millipore, Darmstadt, Germany),4 μM ionomycin and 2.5 μM Fluo-3 AM (Beyotime Institute of Biotechnology, Haimen, China). The toxicities of both drugs and siRNAs were tested using the WST-1 Cell Proliferation and Cytotoxicity Assay Kit (Beyotime), according to the manufacturer’s instructions. At 36, 48, and 60 h post-infection (hpi), DEF cells were collected for subsequent analysis.

### Western blot analysis

Proteins from cells treated with either drugs or siRNAs, or infected with DEV were extracted using immunoprecipitation lysis buffer (Beyotime) with the protease inhibitor phenylmethylsulfonyl fluoride (Beyotime), then boiled for 10 min in 5× loading buffer, separated by 12% sodium dodecyl sulfate-polyacrylamide gel electrophoresis, and transferred onto nitrocellulose membranes (GE Healthcare Life Sciences, Little Chalfont, UK), according to manufacturers’ instructions. The membranes were blocked with 3% bovine serum albumin (Sigma-Aldrich Corporation, St. Louis, MO, USA) for 2 h at room temperature and then incubated with the following primary antibodies for 2 h at room temperature: rabbit anti-LC3B antibody (Sigma-Aldrich Corporation), mMouse anti-CaMKKβ antibody (Sigma-Aldrich Corporation), rabbit anti-p-AMPK antibody (Thermo Fisher Scientific, Waltham, MA, USA), mouse anti-AMPK antibody (Thermo Fisher Scientific), mouse anti-β-actin antibody (Sigma-Aldrich Corporation).Then, the membranes were incubated with IRDye 800 CW goat anti-mouse or goat anti-rabbit immunoglobulin IgG as secondary antibodies for 1 h at room temperature. Antibody detection was conducted using an Odyssey Infrared Fluorescence Scanning Imaging System (LI-COR Biosciences, Lincoln, NE, USA). Quantitation from western blot image intensity was achieved by adding rectangle to the image to gain data directly using the Odyssey Infrared Fluorescence Scanning Imaging System Application Software Version3.0.

### Confocal fluorescence microscopy

For the detection of autophagosomes, DEF cells at 70–80% confluence in culture dishes were transfected with 2.5 μg of the GFP-LC3 plasmid using the Calcium Phosphate Transfection Kit (cat. no. K2780–01; Invitrogen Corporation, Carlsbad, CA, USA). At 24 hpi, chemical-treated or virus-infected DEF cells at different time points were fixed with absolute ethanol for 30 min and the cell nuclei were stained with 4′-6-diamidino-2-phenylindole (cat. no. D1306; Beyotime). The green fluorescence of GFP-LC3 was observed by confocal laser microscopy using a Leica SP2 confocal system (Leica Microsystems, Wetzlar, Germany).

### CaMKKβ siRNA

In order to further study the effects of cell autophagy on viral replication, siRNA targeting the autophagy-related gene beclin-1 was synthesized (Shanghai GenePharma Co., Ltd., Shanghai, China). The sequence of the siRNA was GCC UAC AAC GAG GAC GAU ATT (sense) and UAU CGU CCU CGU UGU AGG CTT (antisense). Six-well plates were transfected with siRNA and negative control (NC)-siRNA using transfection reagents for 24 h and then infected with DEV. Cell samples were collected to detect the effects of siRNA.

### Median tissue culture infective dosed (TCID_50_)

DEF cells were cultured in covered 96-well plates and then infected with DEV virus diluted to 10^− 1^ to 10^− 8^. At 72 hpi, the cells were observed and pathological changes were recorded. Viral titers were determined according to the Reed–Muench method.

### Intracellular Ca^2+^ detection by flow cytometry

Cytosolic free Ca^2+^ ions were detected by using Fluo-3 AM. Fluo-3 AM itself is not combined with Ca^2+^ ions, but once dye is added to the cells, it can hybridize with Fluo-3 AM, and Fluo-3 AM will fluorescence upon binding to Ca^2+^. DEF cells were infected with DEV or treated with BAPTA-AM for the indicated times, then incubated with Fluo-3 AM in the dark at 37 °C for 1 h. Afterward, the cells were suspended in phosphate-buffered saline. To observe fluorescence, as an indicator of intracellular Ca^2+^ ions, the cells were monitored using a flow cytometer (BD FACSAria™; BD Biosciences, San Jose, CA, USA) at an excitation wavelength of 488 nm.

### Statistical analysis

All experimental results are expressed as the mean ± standard deviation (SD) of three independent experiments. The Tukey’s test was used for statistical analysis. A probability (*p*) value of < 0.05 was considered statistically significant.

## Results

### CaMKKβ-AMPK may be involved in DEV-induced autophagy

**Fig. 1:**
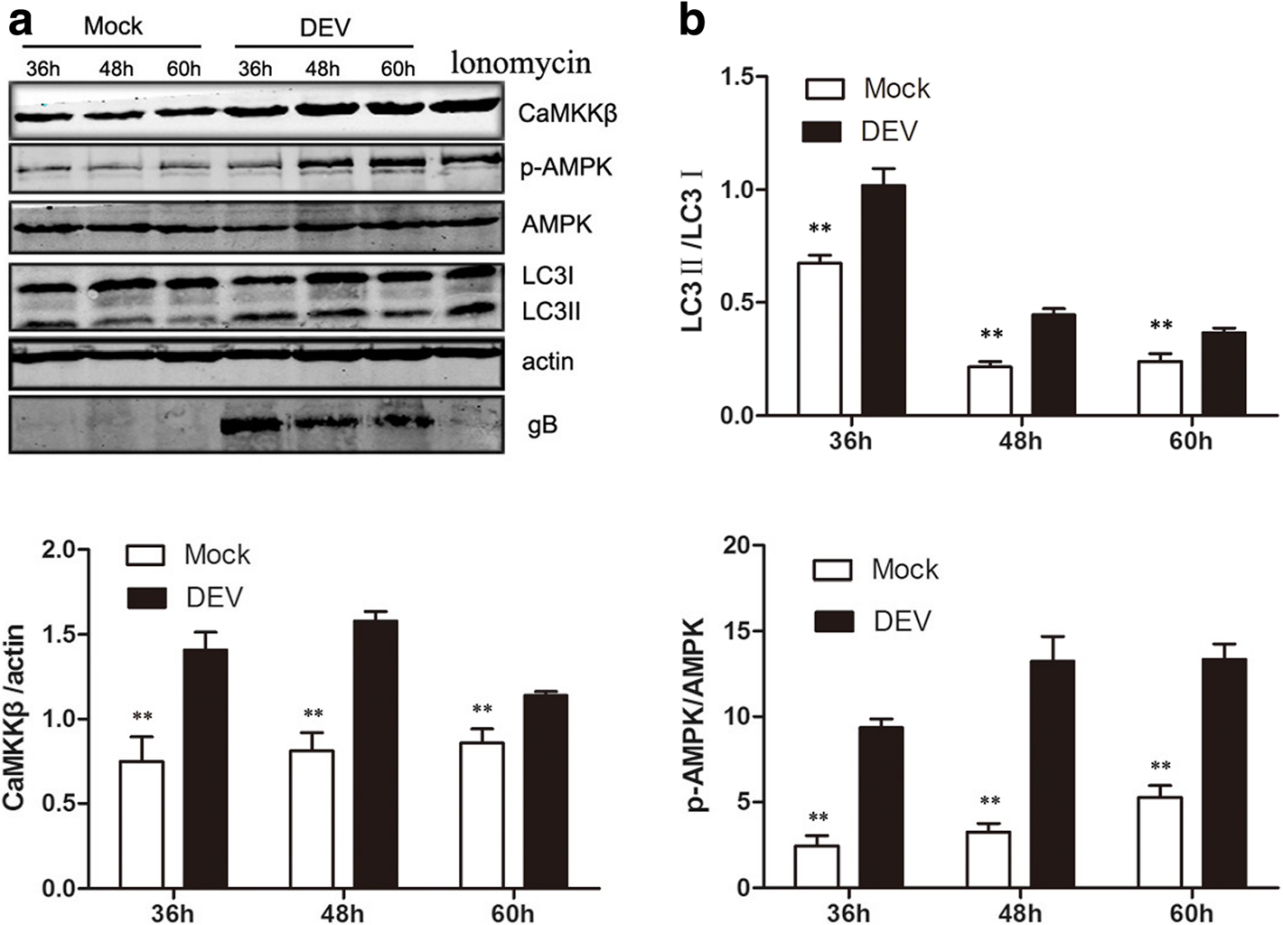
DEV infection activated CaMKKβ and its substrate AMPK, as well as increased the extent of LC3I transformation to LC3II. **a** DEF cells infected with DEV (MOI = 1) or mock-infected cells were lysed and blotted with antibody against CaMKKβ, p-AMPK, AMPK, LC3, and β-actin at the indicated times. Ionomycin treated cells as a postive control (**b**) The ratios of CaMKKβ/β-actin. p-AMPK/AMPK, and LC3II/LC3I in DEF cells from three independent experiments, expressed as means ± SD. *p < 0.05; **p < 0.01

Autophagy of DEV was induced via the AMPK/TSC2/mTOR pathway in DEF cells. An investigation to determine whether any other underlying mechanism of AMPK activation was involved in autophagy induction showed that DEV infection significantly increased intracellular levels of CaMKKβ and its substrate molecule phosphorylated AMPK (p-AMPK) at 36, 48, and 60 hpi, as compared to mock-infected cells. Microtubule-associated protein light chain (LC3) is an autophagy marker protein on the membranes of autophagosomes. When autophagosomes form, LC3I is phosphorylated by phosphatidyl ethanolamine (PE) to LC3II. LC3II remains on autophagosome membranes until fusion with lysosomes. Therefore, to some extent, LC3II expression measures the number of autophagosomes [22]. The expression level of LC3II also was significantly increased (Fig. 1a, b). This result indicated that CaMKKβ-AMPK may be involved in DEV-induced autophagy.

### CaMKKβ is an upstream activator of AMPK involved in DEV-induced autophagy

**Fig. 2:**
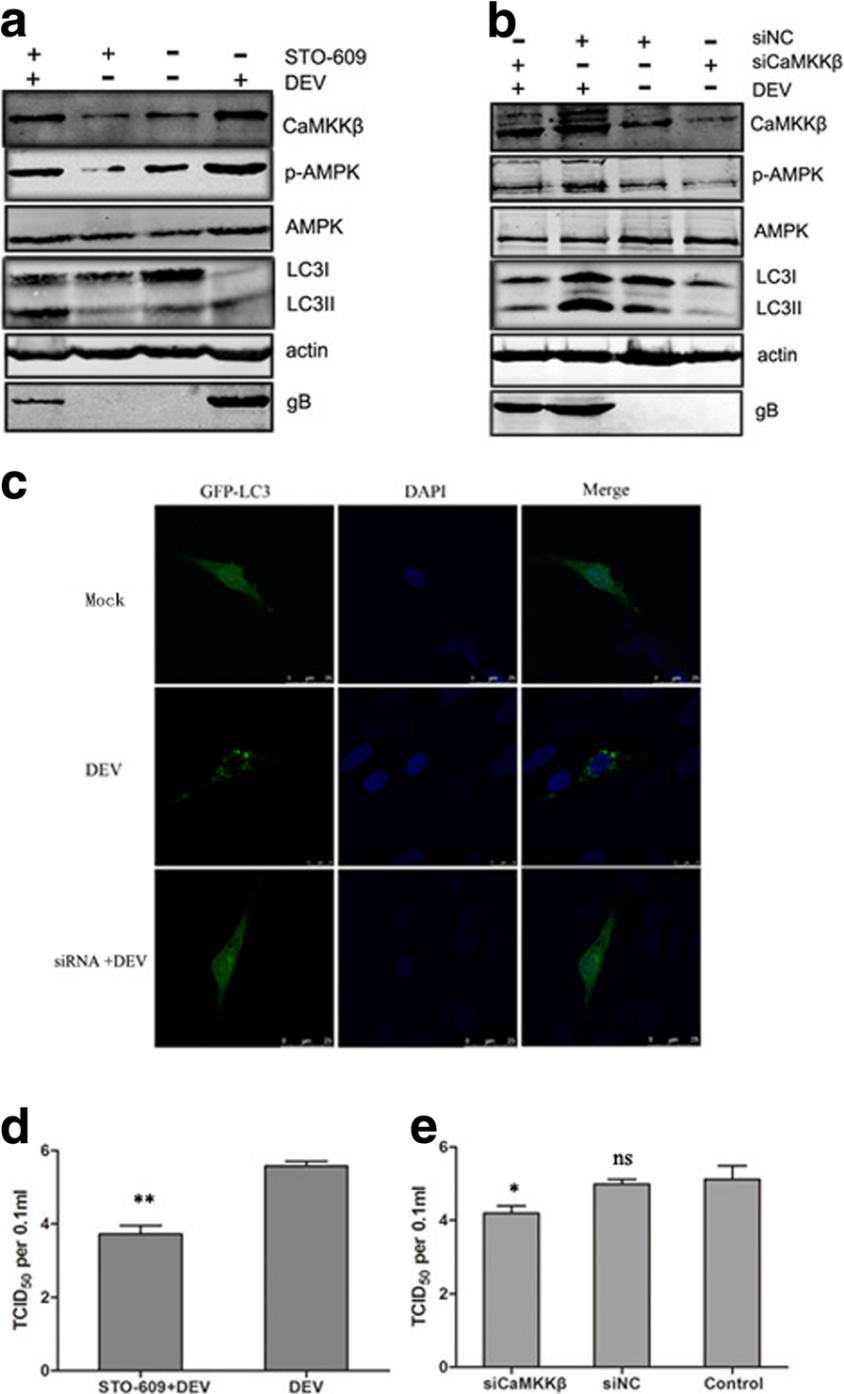
Inhibition of CaMKKβ by drug or siRNA downregulated the activity of AMPK and autophagy. **a** DEF cells were infected with DEV at a MOI of 1 in the presence or absence of STO-609 (10 μM) for 36 hpi. Proteins extracted from cells treated with STO-609 and blotted with antibodies against CaMKKβ, p-AMPK, AMPK, LC3, β-actin and gB. **b** DEF cells were infected with DEV (MOI = 1) in the presence of siRNA targeting CaMKKβ (siCaMKKβ) for the indicated time points. Whole cell lysates at 36 hpi were subjected to western blot analysis. Representative images of immunoblots of target proteins extracted from cells treated with siCaMKKβ and blotted with antibodies against CaMKKβ, p-AMPK, AMPK, LC3, and β-actin. **c** Representative confocal images of DEV-infected DEF cells with or without siCaMKKβ treatment. GFP-LC3 puncta were analyzed. **d, e** Viral titer (TCID_50_) at 48 hpi. All statistical data are reported as the mean ± SEM of three independent experiments (ns, *p* > 0.05; *p < 0.05; and **p < 0.01)

Next, we further verified the role of CaMKKβ in DEV-induced autophagy. Since CaMKKβ is activated in DEV-infected DEF cells, STO-609, a known CaMKKβ inhibitor, was used to assess changes in autophagy corresponding to the inhibition of CaMKKβ. The results showed that activation of AMPK, LC3I transformation to LC3II were significantly lower in DEV-infected cells treated with STO-609, as compared to control cells. In addition, less DEV gB protein was observed in the drug-treated control cells (Fig. 2a).

To eliminate nonspecific effects of chemical drugs, siRNA was used to inhibit CaMKKβ expression. As shown in Fig. 2b, as compared to cells transfected with the NC-siRNA, DEF cells transfected with siRNA, expression levels of CaMKKβ, activated AMPK, and LC3II were significantly reduced (Fig. 2b). The number of GFP-LC3 puncta decreased dramatically in siCaMKKβ -treated DEV-infected cells, as compared to control cells (Fig. 2c).

Due to the decreased expression of viral proteins in response to CaMKKβ inhibition by chemical drugs or siRNA, we further checked whether inhibition of CaMKKβ reduces viral replication. Viral titers were significantly decreased in DEV-infected DEF cells treated with STO-609 or siCaMKKβ. The TCID_50_, as compared to control cells, at 48 hpi is shown in Fig. 2 d, e These results indicated that autophagy is related to CaMKKβ activation in DEV-infected DEF cells, and CaMKKβ is an upstream activator of AMPK involved in DEV-induced autophagy (Fig. 2d, e).

### DEV infection increased intracellular Ca^2+^ content to activate CAMKKβ

**Fig. 3:**
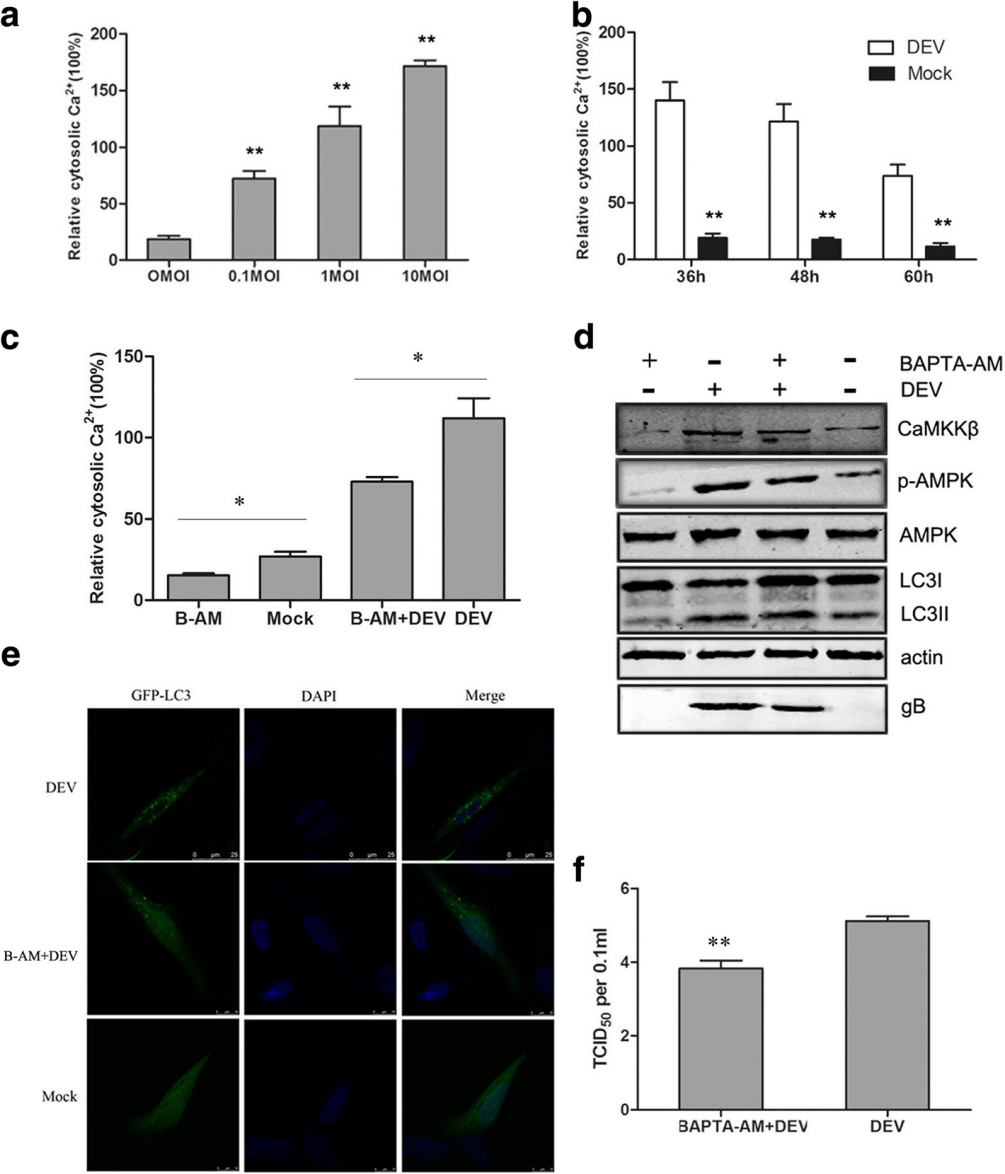
DEV increased cytosolic calcium to activate CaMKKβ and AMPK. **a** DEF cells were infected with DEV at a MOI of 0.1–10. At 48 hpi,the cells cytosolic Ca^2+^ were measured based on Fluo 3-AM, a chemical Ca^2+^ indicator, relative to mock-infected cells. **b** DEF cells were infected with DEV at a MOI of 1. At 36, 48 and 60 hpi, the cells cytosolic Ca^2+^ were measured based on Fluo 3-AM relative to mock-infected cells. **c** DEV-infected cells treated with BAPTA-AM, the cells cytosolic Ca^2+^ were measured based on Fluo 3-AM relative to control cells. **d** Whole lysates of cells treated with BAPTA-AM or DEV collected at 36 hpi were subjected to western blot analysis of CaMKKβ, p-AMPK, AMPK,LC3,β-actin and gB. **e** Representative confocal images of DEV-infected DEF cells with or without BAPTA-AM treatment. GFP-LC3 puncta were analyzed. **f** Viral titer (TCID_50_) at 48 hpi. All statistical data are reported as the mean ± SEM of three independent experiments (ns, *p* > 0.05; *p < 0.05; and **p < 0.01)

In order to further explore mechanisms involved in autophagy induction, upstream regulators were progressively explored. Some reports showed that an increase in cytosolic Ca^2+^ concentration ([Ca^2+^]cyto) promoted the autophagic process [14, 25]. DEV-infected DEF cells were incubated with Fluo-3 AM. Then, at 36, 48 and 60 phi, intracellular Ca^2+^ was detected by flow cytometry, which showed that cytosolic Ca^2+^ in DEV-infected cells was higher than in mock-infected control cells, respectively (Fig. 3b). The result also showed that the increase in cytosolic Ca^2+^ was dependent on the initial viral dose with a DEV multiplicity of infection (MOI) of 0.1–10 at 48 hpi (Fig. 3a).

BAPTA-AM is a well-established chelator of intracellular Ca^2+^ ions. Mock- or DEV-infected DEF cells were treated with 25 μm BAPTA-AM for 30 h, and intracellular Ca^2+^ was detected by flow cytometry. As expected, the results suggested that the addition of BAPTA-AM reduced intracellular Ca^2+^ levels (Fig. 3 c). Accordingly, CaMKKβ and AMPK activities were significantly decreased in BAPTA-AM-treated cells. After the addition of BAPTA-AM, LC3II expression and viral protein synthesis were significantly reduced (Fig. 3d). GFP-LC3 distribution was also observed by a confocal fluorescence microscopy as discrete puncta associated with autophagic vacuoles. The change in GFP-LC3 puncta number after drug treatment may response to changes in autophagic activity. As speculation, the number of GFP-LC3 puncta remarkable decreased in BAPTA-AM-treated DEV-infected cells, as compared to control cells, suggesting the inhibition of autophagy (Fig. 3e).

The expression of gB protein was reduced in BAPTA-AM-treated DEF cells, as compared with control cells. Consistent with the result, virus titers were significantly reduced in BAPTA-AM-treated DEF cells, as compared with control group cells. TCID_50_ assay was used to measure the DEV viral titers (Fig. 3f). Together, these data reflect that Ca^2+^ is necessary for the function of autophagy in DEV-infected DEF cells in a CaMKKβ- and AMPK-dependent process.

### Cell viability unaffected by pharmacological treatment

**Fig. 4:**
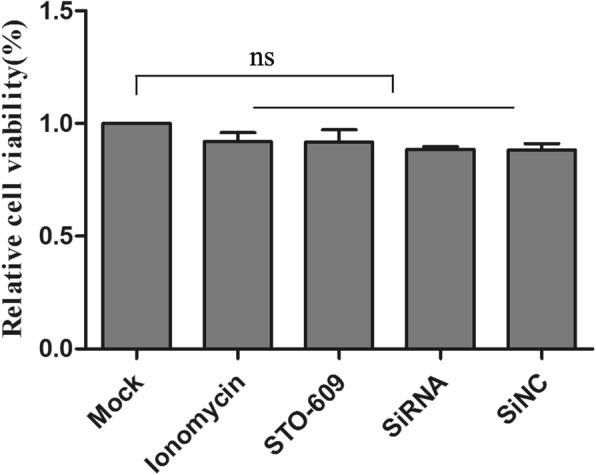
SiRNA or pharmacology had no effect on cell viability. After cells were treated with Lonomycin, STO-609 and siRNA transfection for 48 h, cell viability was tested using WST kits as absorbent density at 450 nm expressed as relative cell viability (ratio of treated to blank cells). Bar represents ± SD; ns indicates no significant difference, p > 0.05

The siRNA targeting of endogenous genes or the drugs might have influenced cell viability and affected our results. The effects on cell viability of compounds used in this study were detected by WST-1 assays. Viability of treated cells was almost equal to mock cells, so the siRNA or pharmacological treatments did not affect DEF cell viability (Fig. 4).

## Discussion

Autophagy is a tightly regulated and evolutionarily conserved intracellular process in which cells destroy and recycle cellular components in lysosomes. Much evidence indicates that virus-induced autophagy plays an important role in the viral life cycle and pathogenicity [26]. Many viruses have been reported to induce autophagy through multiple pathways [6, 27]. The results of our previous study showed that DEV induced autophagic activation through impaired cellular energy metabolism via the AMPK–TSC2–MTOR signaling pathway. Two signaling molecules upstream from AMPK were involved cellular energy and Ca^2+^-mediated CAMKKβ activation. However, it remains unknown whether Ca^2+^-mediated CaMKKβ can activate AMPK and a series of downstream signaling pathways during DEV-induced autophagy. The results of the present study suggested that DEV activates CAMKKβ and its substrate molecule APMK to trigger autophagy in DEF cells by increasing cytosolic Ca^2+^ concentrations.

Autophagy was first associated with intracellular Ca^2+^ regulation. Subsequent studies found that the removal of intracellular or extracellular Ca^2+^ ions inhibited autophagy [28]. Although the association between Ca^2+^ signaling and autophagy regulation has been reported, the underlying mechanisms remain unknown. Ca^2+^ ion control of autophagy is divided into two opposing view, that is, Ca^2+^ ions inhibit autophagy and promote autophagy. In this study, DEV infection induced an increase in intracellular Ca^2+^ and activated the formation of autophagosomes in DEF cells.

CaMKKβ is one of the most potent Ca^2+^-dependent protein kinases and is involved in a variety of signal transduction process. It is well known that AMPK (Thr172), CaMKI (Thr172), and CaMKIV (Thr200) can be directly phosphorylated by CaMKKβ to participate in autophagy [29]. In addition, activation of CaMKKβ is mainly dependent on conformational changes caused by the binding of Ca^2+^ and calmodulin. Therefore, the level of free Ca^2+^ in cytoplasm is essential for the activation of CaMKKβ [30].

Ca^2+^ and CaMKKβ are related with the activation of AMPK in T cells, hypothalamic neurous cells and endothelial cells,implying that Ca^2+^ metabolism may play an important role in AMPK-mTOR-regulated autophagy process [14]. Recent studies have found that in rotavirus-infected cells, CaMKKβ is activated by increased Ca^2+^ levels, further activating AMPK, which leads to subsequent autophagy [18]. Human cytomegalovirus infection can activate the CaMKKβ/AMPK pathway to promote cellular glucose metabolism and viral replication [31]. Here, although DEV infection indeed increased the cytoplasmic content of Ca^2+^ ions, the underlying mechanism is not clear. However, it has been speculated that the virus may encode one or more proteins that change biomembrane permeability to Ca^2+^, causing an increase in [Ca^2+^]cyto originating from ER or Golgi [Ca^2+^] stores, or the extracellular environment [18, 32]. The results of our previous study confirmed that the ER stress response is involved in DEV-induced autophagy, suggesting that there might be some links between ER stress and elevated [Ca^2+^]cyto.

## Conclusions

The results of the present study demonstrated that CaMKKβ is an upstream regulator of AMPK during DEV infection, which contributes to autophagy induction. Activation of CaMKKβ results from an increase in cytosolic Ca^2+^ content. This research lays a foundation for DEV pathogenic mechanism research and provides further insight into DEV–host cell interactions.

## Abbreviations

AMPK: Adenosine 5′-monophosphate-activated protein kinase
BAPTA-AM: 1,2-Bis (2-aminophenoxy) ethane-N, N, N′, N-tetraacetic acid
DEF: Duck embryo fibroblast
DEV: Duck enteritis virus
ER: Endoplasmic reticulum
